# Hepatobiliary langerhans cell histiocytosis mimicking Caroli syndrome and recurring in a liver allograft

**DOI:** 10.3389/fmed.2026.1898715

**Published:** 2026-08-17

**Authors:** Tingting Hu, Chao Wang, Chengjia Liu, Jianzhong Sun

**Affiliations:** Department of Radiology, The Second Affiliated Hospital, Zhejiang University School of Medicine, Hangzhou, China

**Keywords:** allograft recurrence, Caroli syndrome, case report, langerhans cell histiocytosis, liver transplantation

## Abstract

Langerhans cell histiocytosis (LCH) involving the hepatobiliary system can progress to sclerosing cholangitis and cirrhosis and may require liver transplantation (LT). We report a pediatric case in which hepatobiliary LCH was initially interpreted as Caroli syndrome and recurred in the liver allograft after LT. A 4-year-old girl presented with recurrent abdominal pain and imaging findings showing hepatomegaly, multifocal intrahepatic cystic changes, and tortuous bile duct dilatation. A prior needle biopsy supported congenital hepatic fibrosis with focal cirrhosis, and she underwent pediatric orthotopic LT. Explant pathology unexpectedly showed bile duct wall infiltration by S100+, CD1a+, and Langerin+ histiocytes, confirming LCH. Early post-transplant PET-CT showed no definite active extrahepatic disease. Eleven months after LT, while receiving tacrolimus-based immunosuppression, she was readmitted with abdominal pain, and CT, MRI, and PET-CT revealed multiple new hypodense and mildly hypermetabolic hepatic allograft lesions. Allograft biopsy confirmed recurrent LCH, and molecular testing was negative for BRAF V600E. The patient began vincristine and prednisone with Pneumocystis jirovecii prophylaxis; abdominal pain resolved and allograft function remained stable, with partial lesion regression on follow-up MRI. This case illustrates that hepatobiliary LCH may mimic Caroli syndrome before transplantation and can recur in the liver allograft, making complete explant histopathology, functional imaging surveillance, and prompt biopsy essential when new post-LT allograft lesions appear.

## Introduction

Langerhans cell histiocytosis (LCH) is a rare dendritic cell neoplasm defined by clonal accumulation of CD1a- and Langerin-positive histiocytes in affected tissues (1). Bone, skin, and the pituitary gland are common sites of disease, whereas hepatic involvement is a high-risk manifestation that occurs in a minority of pediatric multisystem LCH cases (approximately 15%) (2). Hepatobiliary LCH most often presents with bile duct injury and secondary sclerosing cholangitis, which can progress to cholestasis, biliary cirrhosis, and end-stage liver disease (3). In selected patients with irreversible liver failure, LT remains a rescue option, but recurrence and systemic disease control remain central concerns (4, 5).

A pre-transplant diagnosis of hepatobiliary LCH can be difficult when disease is anatomically confined to the liver and bile ducts. Imaging findings may overlap with congenital or acquired cholangiopathies, including Caroli disease or Caroli syndrome, particularly when intrahepatic bile ducts appear tortuous or cystically dilated (6, 7). This distinction matters because Caroli syndrome is primarily a developmental fibropolycystic disorder, whereas LCH is a clonal histiocytic neoplasm that usually requires systemic assessment and, when active, systemic therapy.

We describe a child who underwent LT for presumed Caroli syndrome and was subsequently diagnosed with hepatobiliary LCH on explant pathology. Eleven months after LT, LCH recurred in the hepatic allograft. The case is unusual because it combines a pre-transplant diagnostic masquerade with biopsy-proven post-transplant allograft recurrence, highlighting surveillance and treatment issues relevant to hepatology, transplantation, oncology, pathology, and imaging.

## Case description

### Patient information and clinical findings

A 4-year-old girl was admitted with recurrent abdominal pain that had first appeared at about 2 years of age and had been intermittently present for approximately 2 years; in the preceding period she had recurrent cholangitis-type episodes that required several courses of intravenous antibiotics at other hospitals. Physical examination at admission showed no obvious abnormality; the abdomen was soft without tenderness, and the liver and spleen were not palpable below the costal margin. On admission (November 2024), the white-cell count was 10.0 × 10^9^/L and the platelet count was 479 × 10^9^/L; the child had mild microcytic anemia (hemoglobin 116 g/L, mean corpuscular volume 73.2 fL). Aspartate aminotransferase was mildly elevated at 47 U/L, C-reactive protein was 18.6 mg/L, and globulin was 30.9 g/L; serum sodium (135 mmol/L), urea (2.30 mmol/L), and creatinine (29.6 micromol/L) were slightly low. Alpha-fetoprotein, blood lipids, bone-metabolism markers, urinalysis, and stool examination were unremarkable, and screening for hepatitis A/B/C/E, HIV, syphilis, and cytomegalovirus/Epstein–Barr virus nucleic acid was negative. At admission, total bilirubin was 28.5 μmol/L (direct 12.3 μmol/L, ≈43% conjugated), albumin 38 g/L, and INR 1.05, with no clinical evidence of hepatic decompensation; anthropometry showed height 100 cm (25th–50th percentile) and weight 15.5 kg (25th percentile) without growth failure. The Pediatric End-Stage Liver Disease (PELD) score calculated at listing was 3 (bilirubin < 2 mg/dL, albumin > 3.5 g/dL, INR < 1.3, no growth failure, no encephalopathy), so listing was driven by the clinical trajectory of recurrent cholangitis, focal cirrhosis on biopsy, and progressive intrahepatic biliary dilatation rather than by biochemical decompensation. A markedly cholestatic profile (gamma-glutamyl transferase 213 U/L, alkaline phosphatase 321 U/L, alanine aminotransferase 132 U/L) was documented in March 2025 after transplantation.

A pre-transplant clinical and serological review did not identify features that would have suggested hepatobiliary LCH over Caroli syndrome. Specifically, the child had no skin or mucosal LCH lesions, no lytic bone lesions, no diabetes insipidus, no lymphadenopathy, and no history of pneumothorax; complete blood count showed a normal white-cell differential, normal eosinophil count, and a platelet count of 479 × 10^9^/L, with no cytopenia suggestive of active marrow infiltration; serum albumin was 38 g/L, INR was 1.05, and the calculated Pediatric End-Stage Liver Disease (PELD) score at listing was 3 (computed 2.75, rounded), consistent with predominantly structural rather than hepatocellular disease. Taken together, these features aligned with cholangiopathy rather than with a systemic histiocytic process, and a pre-transplant diagnosis of LCH was not considered clinically justified.

The available clinical records did not identify relevant family or psychosocial history. The patient had undergone serial hepatobiliary imaging before referral because of suspected congenital cholangiopathy. All patient-specific information was de-identified for publication.

### Relevant past interventions and pre-transplant course

**Figure 1:**
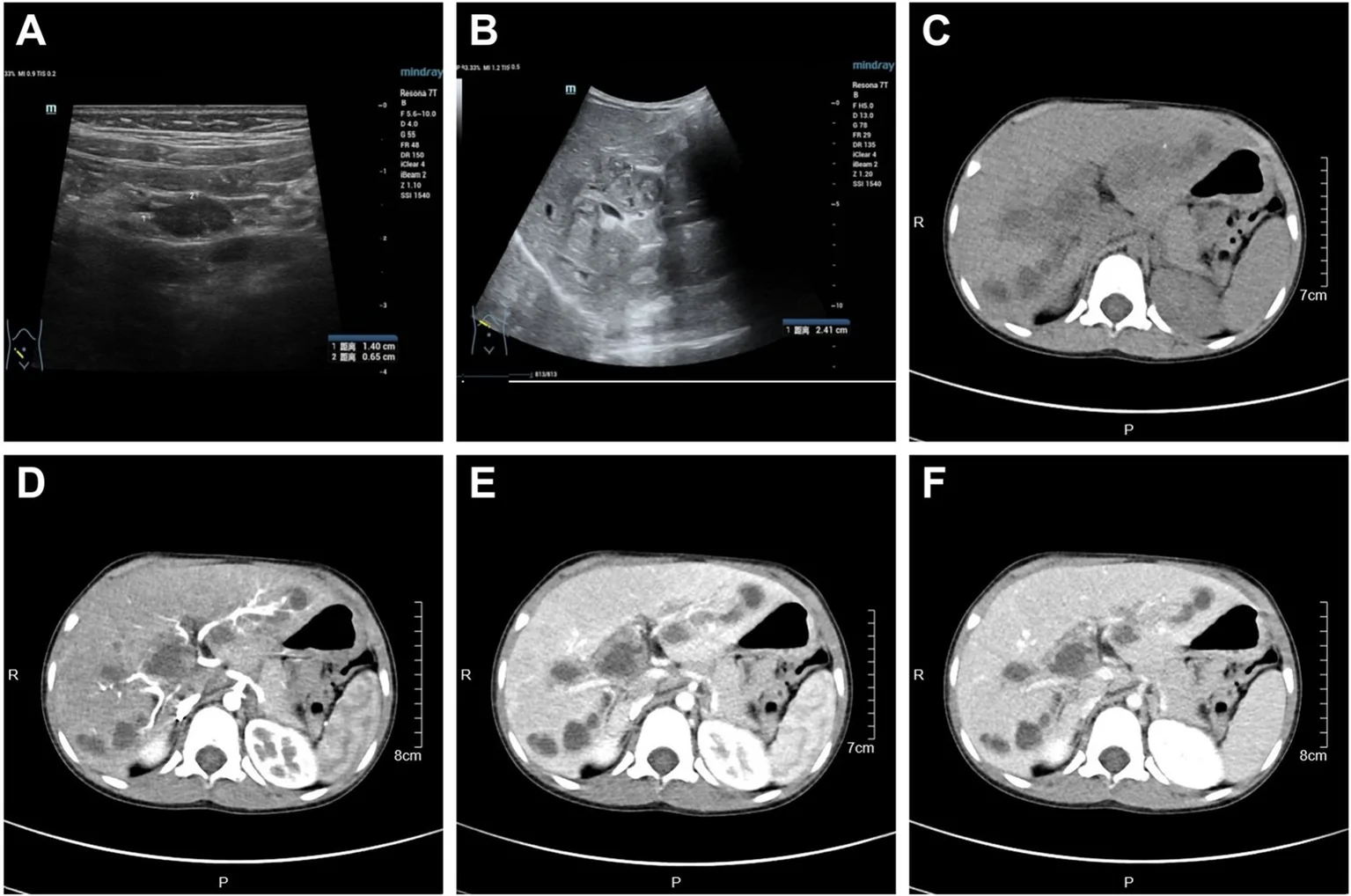
Pre-transplant imaging. **(A,B)** Ultrasonography on 21 November 2024 showed nodular hypoechoic hepatic lesions and multifocal cystic dilatation of intrahepatic bile ducts with wall thickening. **(C–F)** Contrast-enhanced CT on 22 November 2024 showed marked intrahepatic biliary dilatation and enhancing thickened bile duct walls across dynamic phases, supporting the initial radiologic impression of Caroli syndrome.

Pre-transplant abdominal imaging at other hospitals, including ultrasonography, contrast-enhanced computed tomography (CT) with CT angiography, and magnetic resonance cholangiopancreatography (MRCP), showed hepatomegaly with diffuse hepatic lesions, multiple intrahepatic anechoic areas, and tortuous intrahepatic bile duct dilatation with biliary sludge or stones; these findings were repeatedly interpreted as suggestive of Caroli disease. The child also had recurrent cholangitis requiring multiple antibiotic courses. A liver needle biopsy performed at an outside hospital before transplantation showed congenital hepatic fibrosis with focal cirrhosis (immunostaining limited to CD31, CD45, CD68, CK19, CK7, SMA, and Desmin, with negative PAS; CD1a and Langerin were not performed). Liver transient elastography (FibroScan, outside hospital) showed a liver-stiffness value of 5.9 kPa with a controlled attenuation parameter of 184 dB/m. In a 4-year-old child this stiffness value lies at the upper end of the reported normal range (normal median approximately 4.4 kPa; reference range approximately 2.45–5.56 kPa) and therefore indicates only borderline change rather than established cirrhosis; the diagnosis of cirrhosis rested on the ultrasonographic appearance and the needle-biopsy histology rather than on elastography. Because of progressive biliary disease, recurrent cholangitis, and biopsy-proven focal cirrhosis, the patient was listed for pediatric LT. The calculated Pediatric End-Stage Liver Disease (PELD) score at listing was 3 (total bilirubin 28.5 μmol/L, albumin 38 g/L, INR 1.05, no growth failure, no encephalopathy); listing was therefore based on recurrent cholangitis requiring intravenous antibiotics, biopsy-proven focal cirrhosis, progressive intrahepatic biliary dilatation on serial imaging, and a trajectory that was unresponsive to conservative management (Table 1; Figure 1). The MRCP pattern (saccular intrahepatic ductal dilatations with a central dot sign and tortuous, dilated extrahepatic ducts) was characteristic of Caroli syndrome and did not show the irregular pruning, beaded strictures, or narrowed extrahepatic ducts that have been described in hepatobiliary LCH with sclerosing cholangitis (8); the absence of these LCH-typical MRCP features further reduced the prior probability of hepatobiliary LCH in this child.

**Table 1:** Timeline of the episode of care.

Time	Event	Relevant data and outcomes
May–July 2023	Initial hepatobiliary imaging and evaluation	Hepatomegaly, diffuse hepatic lesions, intrahepatic duct dilatation; Caroli syndrome was considered the leading diagnosis. LCH was not suspected at this stage.
July 2024	Liver needle biopsy	Congenital hepatic fibrosis and focal cirrhosis were reported, supporting presumed Caroli syndrome.
21–22 November 2024	Admission and CT/ultrasonography	Recurrent abdominal pain; imaging showed multifocal intrahepatic cystic change and bile duct wall thickening.
February 2025	Orthotopic pediatric liver transplantation	LT was performed for presumed Caroli syndrome with progressive biliary disease and biopsy-proven focal cirrhosis.
13 February 2025	Explant pathology	S100+, CD1a+, and Langerin+ histiocytes confirmed hepatobiliary LCH. At this time the disease was classified as single-system, single-site (liver) LCH involving a risk organ.
March 2025	Post-transplant PET-CT	No definite active extrahepatic LCH was identified.
January 2026	Readmission 11 months after LT	Abdominal pain recurred; CT, MRI, and PET-CT showed multiple new allograft lesions.
24 January 2026	Allograft biopsy	CD1a+, S100+, and Langerin+ recurrent LCH was confirmed; BRAF V600E was negative. Re-staging showed liver and cervical nodal involvement with a negative bone marrow (multisystem, risk-organ-positive disease).
February–April 2026	Vincristine and prednisone	Abdominal pain resolved, allograft function remained stable, and follow-up MRI showed partial lesion regression. The patient received vincristine (1.5 mg/m^2^, administered on 5 and 12 February 2026) rather than vinblastine, plus oral prednisone; the substitution was based on the institutional pediatric-oncology protocol for risk-organ LCH in transplant recipients on calcineurin-inhibitor immunosuppression, in which vincristine replaces vinblastine within a modified LCH-III backbone because of its better-characterized pediatric tolerability profile under tacrolimus co-administration.

In February 2025, the patient underwent orthotopic LT using a whole-liver graft from a pediatric donation after brain death (donation after citizen’s death). The operation was successful, and she received routine postoperative intensive care before transfer to the general ward. Post-transplant immunosuppression consisted of tacrolimus, with trough concentrations adjusted to 4–10 ng/mL, and corticosteroids with gradual tapering.

### Timeline

The episode of care is summarized in Table 1.

## Diagnostic assessment

### Pre-transplant diagnostic challenge and final explant diagnosis

**Figure 2:**
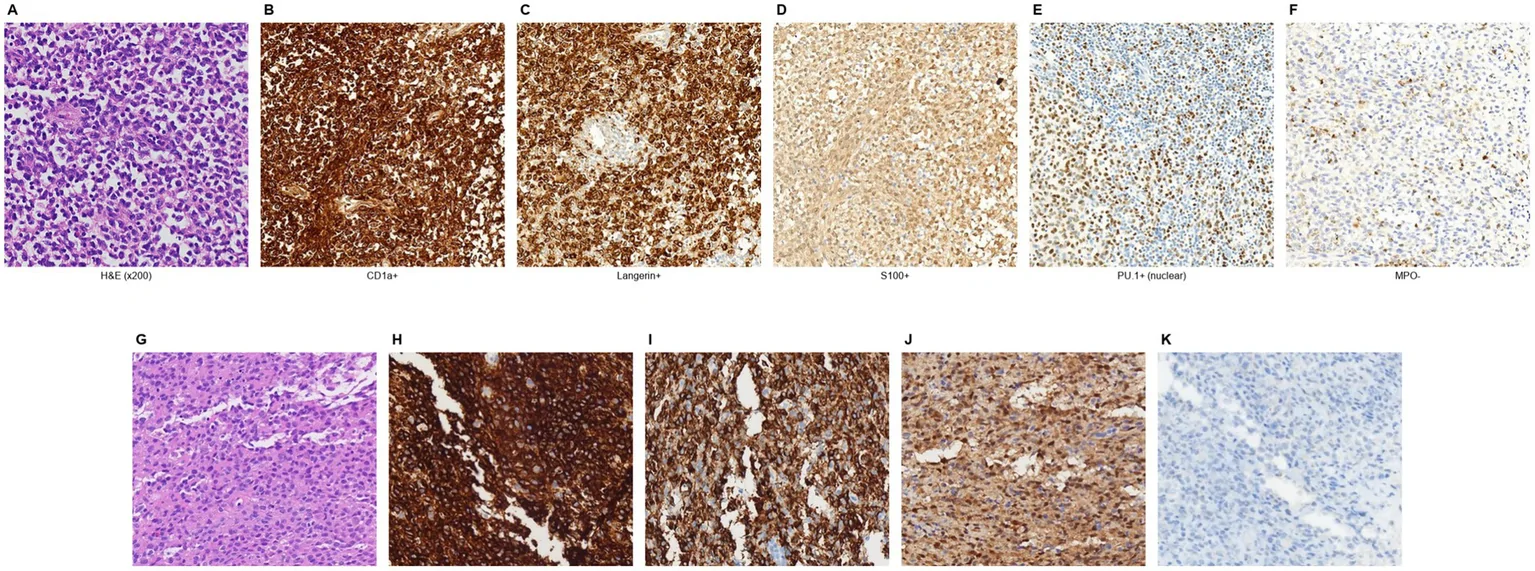
Histopathology of hepatobiliary Langerhans cell histiocytosis (LCH). Top row: Explanted native liver (13 February 2025). **(A)** Hematoxylin and eosin (×200) showing dense histiocyte-like infiltration of the bile-duct walls with admixed lymphocytes. **(B–D)** Immunohistochemistry of the explant showing diffuse positivity for CD1a **(B)**, Langerin/CD207 **(C)**, and S100 **(D)**. **(E)** PU.1 showing diffuse nuclear positivity in the same cells (consistent with a clonal Langerhans-cell process). **(F)** Myeloperoxidase negative (excluding myeloid sarcoma). The Ki-67 proliferation index was approximately 20%. Bottom row: Liver allograft recurrence biopsy (24 January 2026). **(G)** Hematoxylin and eosin (×200) showing histiocyte-like cells admixed with eosinophils. **(H–J)** Immunohistochemistry of the recurrence biopsy showing diffuse positivity for CD1a **(H)**, Langerin/CD207 **(I)**, and S100 **(J)**. **(K)** BRAF V600E negative.

Histopathological examination of the explanted native liver and gallbladder unexpectedly revealed dilated bile ducts with cholestasis and dense infiltration of histiocyte-like cells and sparse lymphocytes in the bile duct walls, together with chronic cholecystitis (Figure 2A). Immunohistochemistry showed diffuse positivity for S100, CD1a, and Langerin. PU.1 showed diffuse nuclear positivity (Figure 2E), whereas myeloperoxidase was negative (Figure 2F). These findings confirmed LCH involving the hepatobiliary system. The Ki-67 proliferation index was approximately 20%; because the prognostic significance of the Ki-67 index in LCH remains uncertain, this value was interpreted with caution (Figures 2B–F).

The diagnostic contribution of CD207/Langerin immunohistochemistry in explanted liver tissue from patients with long-standing cholangiopathy deserves a caveat: reactive Langerin-positive histiocytes can be encountered in the wall of chronically obstructed or inflamed bile ducts (Eminger et al. (9)), so isolated CD207 positivity in the absence of CD1a, S100, or demonstrable clonality should be interpreted with caution. In the present case, this limitation motivated a complete re-examination of the allograft biopsy at recurrence (Figures 2H–K), which confirmed the diagnosis by demonstrating CD1a, Langerin/CD207, and S100 positivity in the same cells, thereby satisfying the immunohistochemical criteria of the Histiocyte Society (10) and the 2022 illustrative update on histiocytic neoplasms (11).

**Figure 3:**
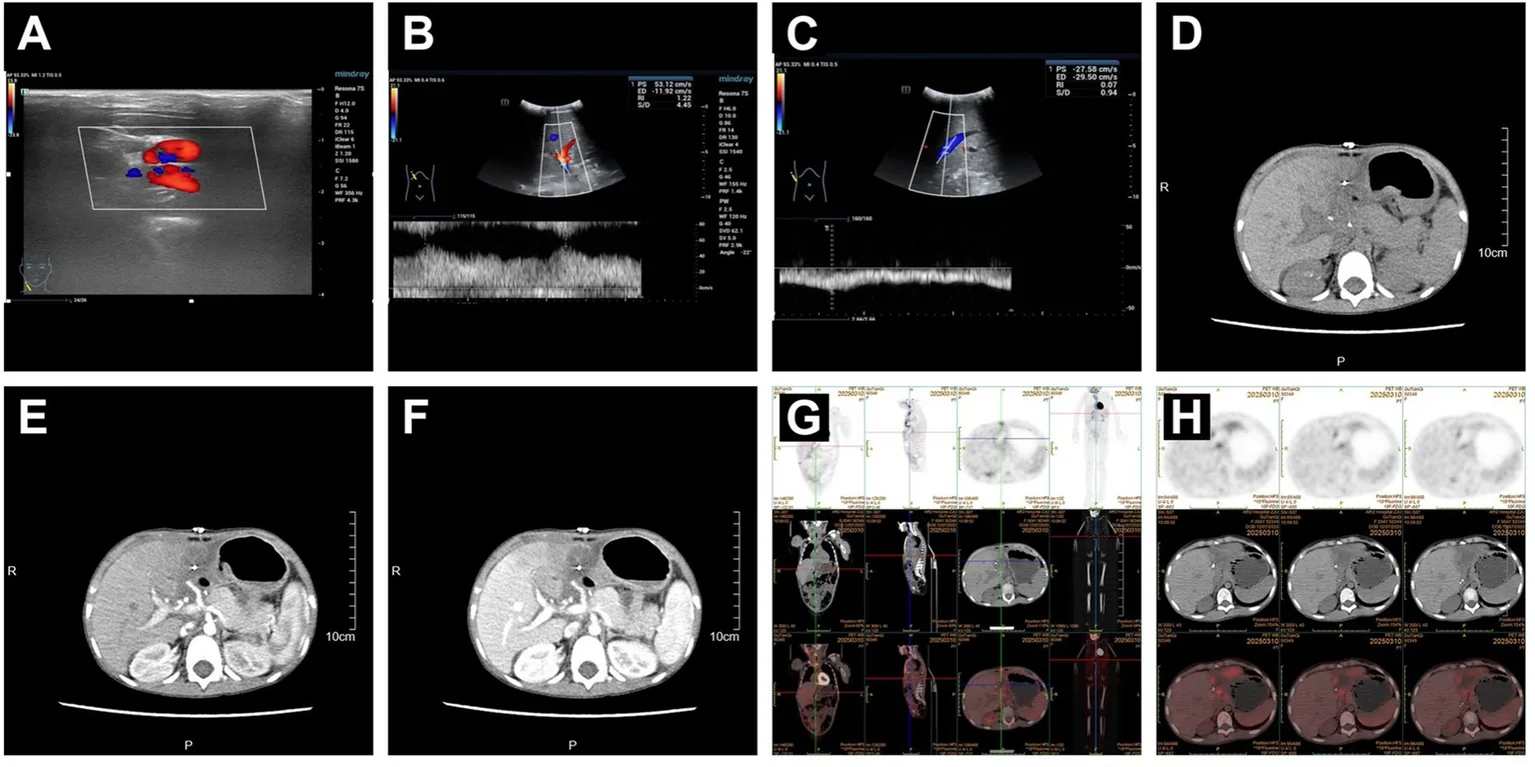
Early post-transplant surveillance imaging. **(A–C)** Post-transplant surveillance ultrasonography on 18 February 2025 showed heterogeneous decreased echogenicity in segment IV of the liver graft without obvious intrahepatic biliary dilatation. **(D–F)** Contrast-enhanced CT on 26 February 2025 showed postoperative fluid and focal low-enhancement change in segment IV, with patent hepatic artery and portal vein and no biliary dilatation. **(G–H)** PET-CT on 10 March 2025 showed mild postoperative metabolic activity and no definite extrahepatic LCH.

After the unexpected diagnosis of LCH, the patient underwent systemic evaluation. Whole-body positron emission tomography-computed tomography (PET-CT) in March 2025 showed mild postoperative reactive inflammatory changes but no definite active extrahepatic LCH (Figure 3).

### Assessment of suspected allograft recurrence

**Figure 4:**
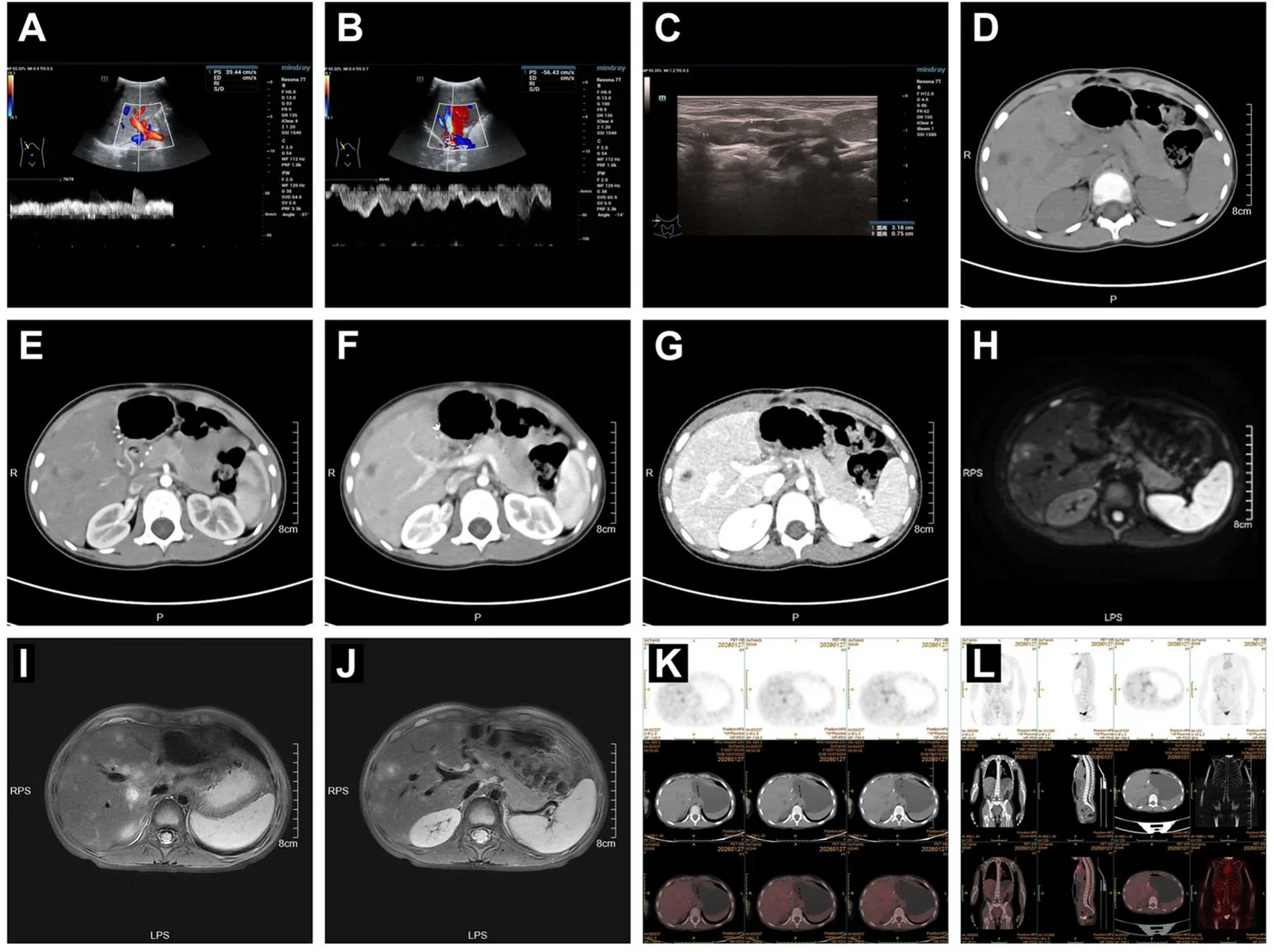
Pre-treatment imaging of allograft recurrence (12–27 January 2026). This imaging constitutes the recurrence work-up performed before salvage chemotherapy (which began in February 2026). **(A–C)** Ultrasonography on 12 January 2026 showed multiple cystic lesions in the liver allograft, some with wall thickening and internal echogenic nodules. **(D–G)** Contrast-enhanced CT on 22 January 2026 showed multiple round low-density hepatic lesions with transient perilesional and rim enhancement. **(H–J)** Enhanced MRI on 24 January 2026 showed multiple long T1/long T2 nodular lesions with partial DWI hyperintensity and peripheral enhancement. **(K, L)** PET-CT on 27 January 2026 showed heterogeneous increased hepatic glucose metabolism, supporting suspected allograft recurrence. Post-chemotherapy follow-up imaging (February–April 2026) is described in the text.

The patient was followed in the outpatient clinic. In January 2026, 11 months after LT, she was readmitted because of an acute exacerbation, with a 1-day history of worsening abdominal pain superimposed on chronic intermittent pain. Laboratory testing showed C-reactive protein of 22.7 mg/L and leukocytosis. Contrast-enhanced abdominal CT and magnetic resonance imaging (MRI) revealed multiple new low-density lesions throughout the liver allograft. PET-CT showed multiple hypodense lesions with slightly increased and heterogeneous glucose metabolism, predominantly in the right hepatic lobe, raising concern for LCH recurrence. New, hypermetabolic, enlarged lymph nodes were also seen in both cervical regions, suggesting probable multisystem involvement; these nodes were not biopsied, and nodal LCH was inferred from PET-CT rather than confirmed histologically (Figure 4).

Ultrasound-guided liver allograft needle biopsy was performed on 24 January 2026. The biopsy showed infiltration by histiocyte-like cells and eosinophils (Figure 2G). Immunohistochemistry was positive for CD1a, S100, and Langerin, and molecular testing was negative for BRAF V600E (Figures 2H–K), confirming recurrent LCH in the transplanted liver. A concurrent bone marrow biopsy showed active trilineage hematopoiesis with no immunohistochemical evidence of tumor involvement (CD1a-, Langerin-, S100-negative). The recurrence was therefore classified as multisystem LCH with liver and cervical nodal involvement and a negative bone marrow.

## Therapeutic intervention, follow-up, and outcomes

### Salvage chemotherapy and clinical outcome

A multidisciplinary tumor board recommended systemic chemotherapy. After contraindications were excluded, the patient began chemotherapy in February 2026 with vincristine (administered on 5 and 12 February 2026) and oral prednisone, together with trimethoprim-sulfamethoxazole for Pneumocystis jirovecii prophylaxis. Vincristine, rather than the standard front-line vinblastine, was used in this patient because the institutional pediatric-oncology protocol for risk-organ LCH in children receiving calcineurin-inhibitor-based immunosuppression adopts a modified LCH-III backbone in which vincristine replaces vinblastine; the substitution was recorded in the multidisciplinary tumor-board decision and was consistent with vincristine’s better-characterized pediatric tolerability profile when co-administered with tacrolimus. The patient tolerated initial chemotherapy cycles, abdominal pain resolved, and liver allograft function remained stable at discharge. Follow-up ultrasonography and MRI between February and April 2026 showed persistent hepatic lesions with mixed interval changes and partial regression on the most recent MRI.

The available records did not document severe chemotherapy-related adverse events during the reported follow-up. The patient remained under joint monitoring by the transplant and oncology teams. Longer follow-up is needed to determine the durability of disease control and allograft survival.

## Discussion

This case illustrates two clinically relevant features of hepatobiliary LCH: it can closely mimic Caroli syndrome before transplantation, and it can recur within a liver allograft after LT. Hepatobiliary involvement in LCH often manifests as sclerosing cholangitis and can progress to cirrhosis despite systemic therapy (8). Caroli syndrome, in contrast, is a congenital fibropolycystic disorder characterized by multifocal cystic dilatation of the intrahepatic bile ducts, often associated with congenital hepatic fibrosis (12). In our patient, the combination of tortuous intrahepatic bile duct dilatation on imaging and congenital hepatic fibrosis on pre-transplant needle biopsy strongly supported the initial diagnosis of Caroli syndrome. The final diagnosis became clear only after complete examination of the explanted liver showed CD1a+, S100+, and Langerin+ histiocytic infiltration of the bile duct walls. A similar diagnostic pitfall has been reported in a pediatric LCH case initially interpreted as Caroli disease (7).

The case also underscores the limits of needle biopsy in diffuse biliary or periportal disease. Sampling may capture fibrosis and ductal remodeling while missing focal histiocytic infiltration. Because LCH is frequently chemo-responsive, the central counterfactual raised by this case is whether earlier recognition could have averted or deferred transplantation. In our patient the pre-transplant needle biopsy was performed at an outside hospital, and its immunohistochemical work-up was confined to markers directed at congenital fibropolycystic disease (CD31, CD45, CD68, CK19, CK7, SMA, and Desmin, with PAS); CD1a and Langerin were not applied, and the archived material was not subsequently re-stained for these markers after the explant diagnosis, so it remains unknown whether a histiocytic infiltrate was already present but unsought. This limitation is particularly important when imaging suggests a congenital cholangiopathy but the clinical course is progressive or atypical. In such settings, repeat biopsy, expanded immunohistochemistry including CD1a and Langerin, and systemic evaluation may be useful before LT if the patient’s condition allows. Once LT has been performed, explant pathology should be examined carefully, because the explant may reveal an occult neoplastic or inflammatory process that changes post-transplant surveillance and treatment.

LT is an accepted rescue therapy for advanced hepatic LCH when irreversible liver failure has developed (4, 5, 13). In the population-based analysis and systematic review by Ziogas et al. (4), LT for LCH achieved acceptable outcomes but post-transplant disease recurrence was reported in a substantial proportion of patients, underscoring that transplantation removes the affected organ but not the underlying clonal disease. This framing is central to interpreting the present case. LCH is a clonal systemic neoplasm, and this child received no LCH-directed systemic therapy before or after transplantation; a single negative early post-transplant PET-CT does not establish eradication of disease. The event at 11 months may therefore reflect persistence and progression of inadequately treated systemic disease rather than a true *de novo* recurrence driven by immunosuppression. This interpretation is arguably the more parsimonious explanation and is presented here as the primary hypothesis. A complementary, non-exclusive possibility is that calcineurin-inhibitor-based immunosuppression altered immune surveillance: Honda et al. (14) described LCH after living donor LT under tacrolimus-based immunosuppression, whereas other reports describe tacrolimus-containing regimens, sometimes with chemotherapy or basiliximab, without early recurrence (15, 16). These reports do not establish causality, and we have tempered our previous emphasis on immunosuppression accordingly. Together they support systemic staging, LCH-directed therapy where indicated, and close monitoring when LCH is identified before or after LT.

In the present case, recurrence occurred 11 months after LT during tacrolimus therapy, with therapeutic trough levels documented during follow-up. The recurrence was not clinically silent: the patient developed abdominal pain, and cross-sectional imaging showed multiple new allograft lesions. PET-CT was useful because it demonstrated heterogeneous metabolic activity within the hepatic lesions and identified new cervical lymphadenopathy. However, imaging alone could not distinguish recurrent LCH from infection, post-transplant lymphoproliferative disease, ischemic lesions, or other inflammatory processes. Allograft biopsy was therefore essential. The biopsy confirmed recurrent LCH and excluded BRAF V600E, guiding the decision to use conventional chemotherapy rather than BRAF-targeted therapy.

The treatment response in this case is consistent with prior experience that post-LT LCH recurrence can be controlled when recognized promptly. Hadzic et al. (17) reported intrahepatic graft recurrence after pediatric LT that responded to conventional chemotherapy. Our patient similarly received vincristine and prednisone, with resolution of abdominal pain, stable graft function, and partial radiologic regression. This outcome supports a practical management strategy for similar cases: new allograft lesions in a patient with known or newly recognized LCH should prompt functional imaging, tissue confirmation, molecular profiling, and multidisciplinary adjustment of oncologic and transplant immunosuppressive treatment.

This report has limitations. It describes a single patient, so it cannot define the incidence of allograft recurrence or the causal effect of tacrolimus on LCH relapse. Follow-up after salvage chemotherapy remains relatively short, and longer observation is needed to determine durability of disease control and graft survival. In addition, molecular characterization was limited to BRAF V600E. A negative BRAF V600E result does not exclude MAPK-pathway activation, which in LCH is frequently driven by MAP2K1 mutations, non-V600E BRAF alterations, ARAF, and other potentially targetable events (18). This limitation strengthens the rationale for more comprehensive sequencing in recurrent disease, because identification of an actionable alteration could open an opportunity for targeted therapy in selected patients.

The main take-away lesson is that hepatobiliary LCH can resemble Caroli syndrome before transplantation and may recur in, or persist within, the allograft after LT. Complete explant histopathology is therefore important when LT is performed for presumed cholangiopathy, and new post-LT allograft lesions should prompt functional imaging when appropriate, early biopsy confirmation, molecular testing, systemic re-staging, and multidisciplinary treatment planning. Because LCH is a systemic clonal disease, attention to LCH-directed therapy, and not transplantation alone, is essential.

### Diagnostic uncertainty–isolated hepatobiliary LCH and its mimicry of Caroli syndrome

Hepatic LCH without involvement of any other organ system is exceptional; in the largest pediatric cohort to date (Ge et al., (2)), liver involvement was invariably accompanied by at least one additional risk-organ site. The present case therefore calls for explicit diagnostic humility. Three sources of uncertainty warrant mention. First, the 24 July 2024 native-liver needle biopsy was reported as showing congenital hepatic fibrosis and focal cirrhosis without prospective Langerin/CD1a staining, so the absence of LCH features at that time cannot be fully excluded by sampling. Second, the explant IHC (Figures 2B–F) showed not only Langerin/CD207 positivity but also diffuse CD1a, S100, and nuclear PU.1 positivity in the same cells, with negative myeloperoxidase and a Ki-67 proliferation index of approximately 20%; this combination (CD1a+/Langerin+/S100+/PU.1+/MPO-, Figures 2B–F) is essentially pathognomonic of Langerhans cell neoplasia and effectively excludes both reactive histiocytosis (in which PU.1 is only weakly or focally expressed, Figure 2E) and myeloid sarcoma (which would be myeloperoxidase-positive, Figure 2F) (see caveat in Pre-transplant diagnostic challenge). Third, no molecular confirmation of clonality (BRAF V600E, MAP2K1, or other MAPK-pathway alterations) was obtained at either biopsy. Under the 2022 illustrative Histiocyte Society framework, the present case is best classified as a non-clonally-verified, single-system, single-site (liver) LCH with risk-organ involvement. The alternative – reactive histiocytic infiltration of the bile-duct wall in the context of Caroli-related cholestasis – is considered unlikely but cannot be definitively excluded, and the recurrence biopsy (Figures 2G–K) with full CD1a/Langerin/S100 positivity and the same pattern at the allograft site provides the strongest available evidence that the disease was a clonal LCH from the outset.

### Mechanistic considerations for the post-transplant LCH-like liver recurrence

Two mechanisms have been proposed for LCH recurrence in a liver allograft: donor-derived LCH (transmitted with the graft) and recipient-derived reactivation of a residual histiocytic clone that was not eradicated by transplantation. In the present case, the whole-liver graft was procured from a donation-after-brain-death pediatric donor, and the graft biopsy 6 weeks after transplant showed no histiocytic infiltrate (Figure 3); the first allograft lesion was identified only at month 11 (Figure 4). The temporal and histologic pattern therefore favor recipient-derived reactivation. The role of tacrolimus in this reactivation deserves comment. Calcineurin inhibition can blunt regulatory T-cell expansion, and an mTOR-inhibitor switch has been reported to coincide with clinical LCH reactivation in some pediatric recipients (Hadzic et al. (17); Honda et al. (14)). A unified interpretation is that residual recipient-derived BRAF-wildtype Langerhans cell precursors may persist in bone marrow or lymphoid reservoirs; once T-cell surveillance is altered by calcineurin inhibition, and especially after a switch in the immunosuppressive class, the latent clone can expand and seed the allograft. This framework, although not directly proven in the present child, is consistent with the 11-month latency, the BRAF V600E-negative molecular profile, and the favorable response to re-introduction of cytoreductive chemotherapy while immunosuppression was continued.

### Clinical implications for hepatobiliary LCH and post-transplant surveillance

Three clinical implications follow. First, when LT is contemplated for presumed Caroli syndrome in a child, the pre-transplant workup should include targeted immunohistochemistry (CD1a, Langerin, S100) on the most recent liver biopsy, and any CD207-positive infiltrate should be corroborated with a second marker before discounting hepatobiliary LCH (19, 20). Second, post-LT surveillance in children with LCH-related liver disease should include a low threshold for cross-sectional imaging and protocol allograft biopsy when new lesions appear, because non-invasive imaging alone may underestimate both disease burden and histologic activity. Third, switching the maintenance immunosuppressant from a calcineurin inhibitor to an mTOR inhibitor in this specific population should be considered cautiously and, when necessary, accompanied by a structured LCH-surveillance plan (cross-sectional imaging, BRAF-status monitoring when tissue becomes available, and a low threshold for allograft biopsy). These implications are derived from a single case and require confirmation in larger registries such as the Histiocyte Society LCH-IV follow-up cohort.

## Patient perspective

A direct patient-authored perspective was not available because the patient was a young child. The clinical record documented resolution of abdominal pain after chemotherapy and clinical stability at discharge. The patient’s parent or legal guardian provided written informed consent for publication.

## Data Availability

The original contributions presented in the study are included in the article/supplementary material, further inquiries can be directed to the corresponding author.
